# Continue the Scaling of Electronic Devices with Transition Metal Dichalcogenide Semiconductors

**DOI:** 10.1021/acs.nanolett.4c06007

**Published:** 2025-02-24

**Authors:** Fangyuan Zheng, Wanqing Meng, Lain-Jong Li

**Affiliations:** Department of Mechanical Engineering, The University of Hong Kong, Hong Kong, China

**Keywords:** future transistors, scaling, transition metal dichalcogenide, electrical properties, silicon

## Abstract

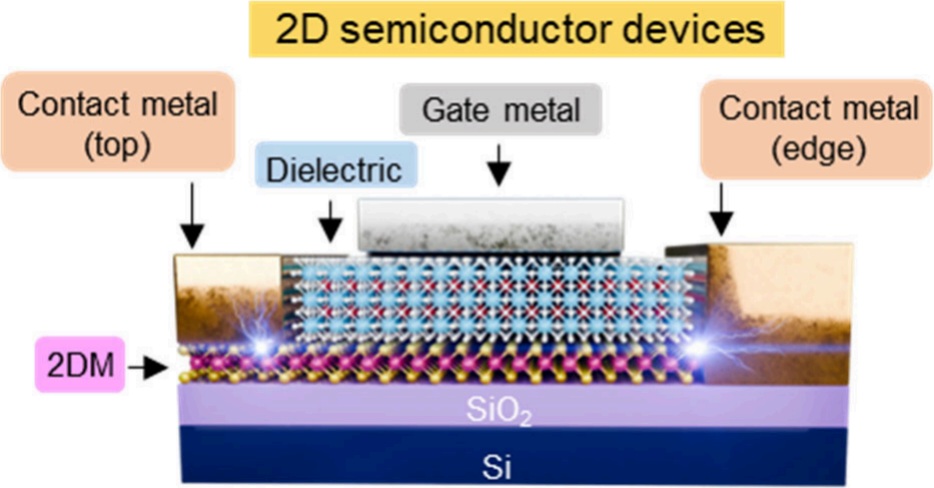

Two-dimensional (2D) transition metal dichalcogenide (TMD) semiconductors have emerged as promising candidates for further device scaling. However, the full potential of 2D TMD materials in advanced electronics remains to be explored. In this Mini-Review, we discuss the trends in modern transistor technology and the issues of silicon (Si) moving toward subnanometer technology nodes, highlighting the prospects of 2D materials in overcoming the limitations of conventional Si-based metal-oxide-semiconductor field-effect transistors (MOSFETs). We focus on the growth and characterization techniques crucial to assessing the quality and electrical properties of 2D TMD materials. The technical challenges that significantly affect the performance of 2D transistors, such as contact resistance, dielectric scaling, and device architecture, are also discussed. The potential benefits and perspectives of utilizing 2D materials are summarized based on the recent simulation results for both devices and circuits.

## 1 Trends in Device Technology

### 1.1 Evolution of Si Transistors

**Figure 1:**
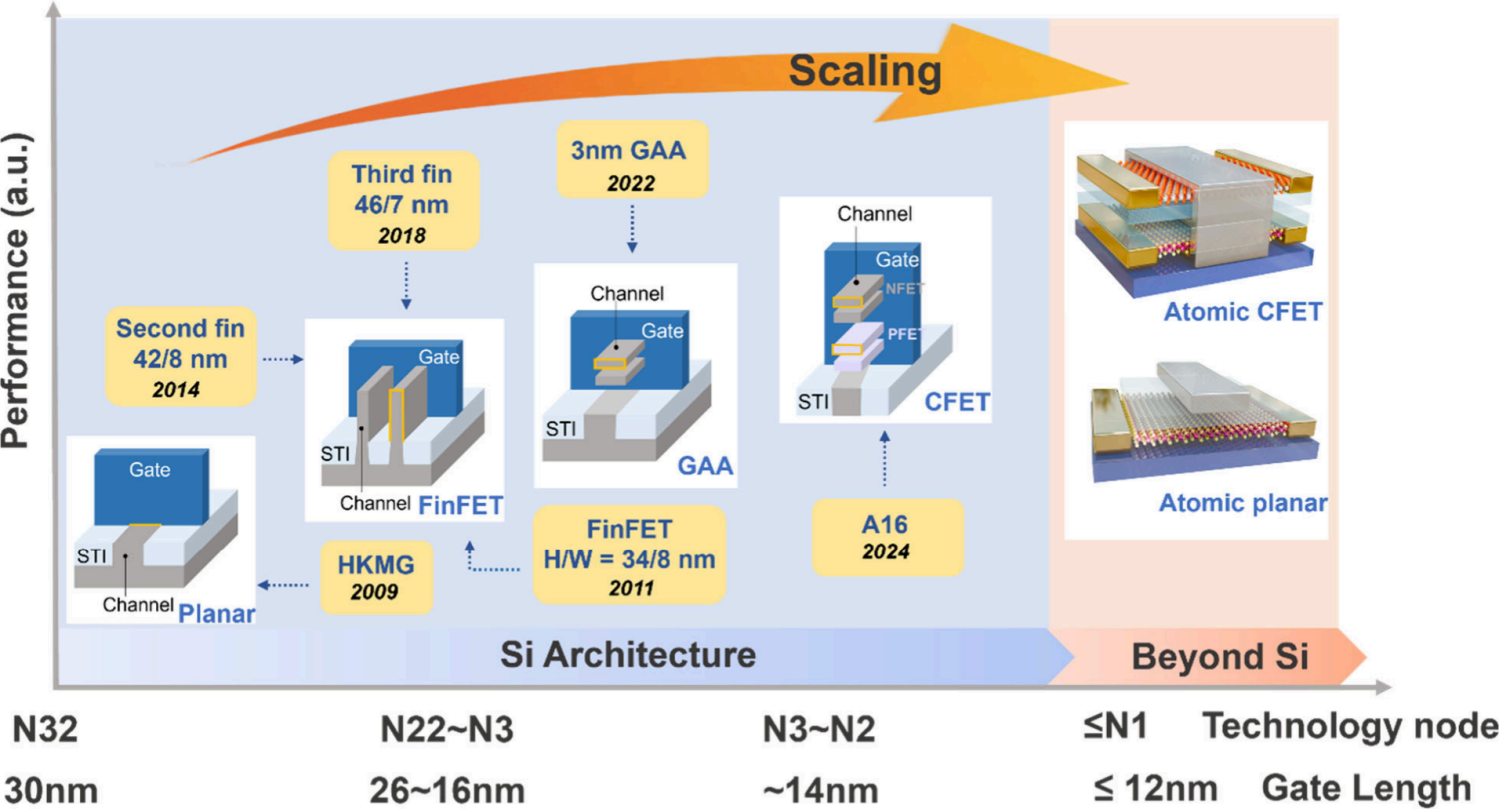
**Trend of transistor scaling versus technology node and gate length.** The *x*-axis presents the technology nodes and corresponding gate length developing with scaling, and the *y*-axis shows the performance increase. The materials innovation introduced high-κ dielectrics with metal gates (HKMG) in 2009. Architecture developed from planar to FinFET in 2011 and recent evolution to GAA, followed by next-generation GAA-CFET. 2D materials- or CNT-based NS FETs are expected to carry on the device scaling for improving performance at the stage beyond N1.

Traditional field-effect transistors (FETs) are primarily based on bulk or three-dimensional (3D) silicon (Si) semiconductor channels. Over the past several decades, devices have been successfully shrunk to nanoscale dimensions in accordance with Moore’s law.^1^ Scaling has brought significant benefits, such as increased ON current and reduced footprint, ultimately improving the speed and lowering the power consumption per transistor. However, scaling also introduces challenges, including increased gate and source–drain leakage as well as a loss of gate control. To this end, the semiconductor industry has developed advanced transistor structures. Technology nodes and gate lengths are two critical parameters, representing the minimum feature size of a transistor and the physical distance between the source and drain regions, respectively. Key technology nodes and gate lengths and their corresponding structures, as illustrated in Figure 1, demonstrate the progression from planar FETs to FinFETs and then to gate-all-around (GAA) FETs. Further advancements include complementary FETs based on GAA (GAA-CFETs) and non-Si nanosheet (NS) FETs. The evolution of transistor structures and materials aims at enhancing performance and lowering power consumption per transistor for more compact electronics.

### 1.2 Issues with Si

Nevertheless, further scaling of transistors is still challenging, as maintaining optimal device performance requires substantial reductions in channel length. The ultimate gate length may approach the 12 nm range at the 1 nm technology node (N1) for FETs. Yet the inherent nature of Si unavoidably introduces surface roughness and defects in reaching the required nanoscale thickness. The unavoidable surface scattering in 3D Si channels significantly reduces the carrier mobility due to the enhanced interaction between charge carriers and the carriers in the thin Si channels. Furthermore, advanced Si structures, such as FinFETs and GAA, are associated with challenges stemming from the heightened gate and drain capacitance caused by their intricate 3D design. Complicated Si structures introduce significant parasitic capacitance in circuits and increase wire resistance from their tiny and dense metal interconnects. This, as a result, exacerbates resistive–capacitive (RC) delays, which decrease the transistor’s switching speed and energy efficiency, ultimately decreasing circuit performance.^2^

### 1.3 2D Materials on the Transistor Roadmap

**Figure 2:**
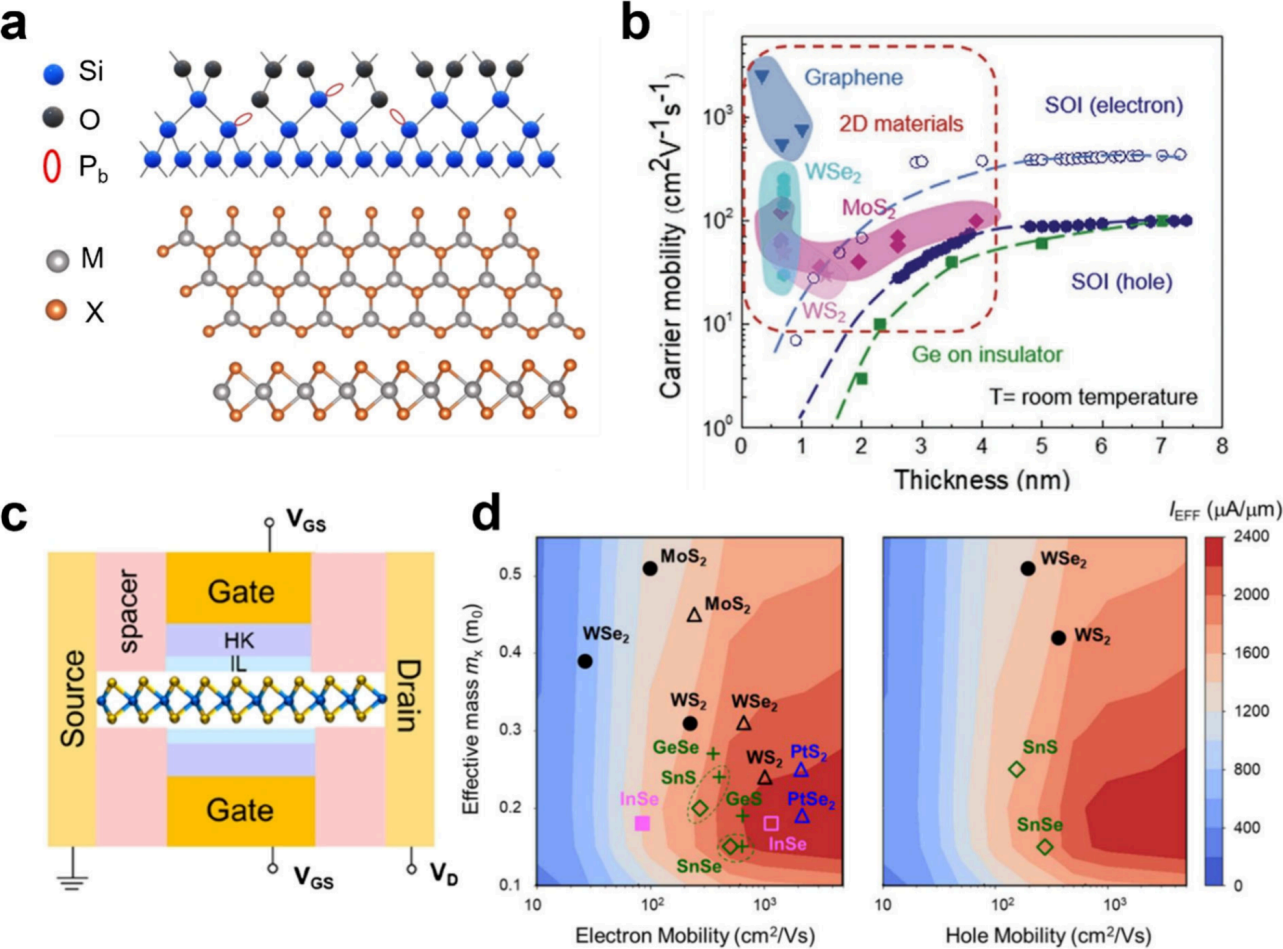
**2D materials compared with 3D materials for FETs. a.** Atomic structures of Si (up) and 2D TMDs (down). Ultrathin Si contains dangling bonds or exhibits bonding to heteroatoms (such as oxygen), while 2D TMDs have a pristine surface with saturated bonding. **b.** Carrier mobility as a function of channel thickness scaling at room temperature (graphene, MoS_2_, WS_2_, and WSe_2_). 2D TMDs provide smaller channel thicknesses and higher electron mobilities compared to Si and Ge at sub-1 nm. Reproduced with permission from ref (5). Copyright 2022 John Wiley and Sons. **c.** Device structure of a monolayer 2D material with a dual-gate setup comprising top and bottom gates. Reproduced with permission from ref (8). Copyright 2021 John Wiley and Sons. **d.** The contour map displays the I_EFF_ based on the mass and mobility of electrons/holes in monolayer 2D materials. Filled symbols represent mobility by the Boltzmann transport equation, with electron–phonon coupling matrix elements from first-principles calculations, while open symbols use the Takagi formula, with effective deformation potential and mass extracted from first-principles calculations. Reproduced with permission from ref (8). Copyright 2021 John Wiley and Sons.

The smooth surface and saturated surface bonding in two-dimensional (2D) materials present the possibility of achieving superior channel-to-dielectric interfaces. Meanwhile, the ultrathin thickness of monolayer 2D materials is beneficial for maintaining gate controllability upon extreme scaling. Since graphene’s exfoliation in 2004,^3^ various other 2D materials have been explored as promising candidates in electronics, with transition metal dichalcogenides (TMDs) forming a substantial group among them. TMDs are represented by a general formula of MX_2_, where M is a transition metal (e.g., Mo or W) and X is a chalcogen (e.g., S, Se, or Te) (Figure 2a). They possess a notable advantage over graphene because of their extensive bandgap tunability, which can be achieved through composition, thickness, and strain control.^4^ Certain 2D TMDs, including MoS_2_, WSe_2_, and WS_2_, exhibit markedly higher electron mobility than Si with a comparable thickness for advanced nodes (Figure 2b).^5^ Despite these promising characteristics, 2D materials are still in the early stages of development for transistor applications, and further research is needed to unlock their full potential.

## 2 2D Materials for Electronics

### 2.1 Selection of Materials

The potential of TMDs to act as channel materials (n- and p-channels) in metal-oxide-semiconductor field-effect transistors (MOSFETs) has gained considerable interest. For instance, monolayer MoS_2_ has a direct bandgap (*E*_g_) of 2.4 eV and a moderate electron effective mass (*m*_e_) of 0.57*m*_0_ for nFETs.^1^ Meanwhile, monolayer WSe_2_, with a bandgap (*E*_g_) of 2.01 eV and a hole effective mass (*m*_h_) of approximately 0.35*m*_0_, is considered as a candidate for pFETs^6^ (Table 1). Other 2D TMDs, such as MoSe_2_, WS_2_, and PtSe_2_, have also attracted significant attention. To effectively select promising materials for transistors, simulations predict the electrical performance of the different materials. Figure 2c displays the proposed device configuration, showing a monolayer 2D material with a dual-gate setup comprising top and bottom gates for optimal electrostatics. Theoretical modeling has been applied to potential 2D semiconductor materials to predict their on-state current in a 7 nm gate length transistor. Figure 2d shows the calculated effective drive current, *I*_EFF_,^7^ as a function of the effective mass and electron/hole (left/right) mobilities along the transport direction *X*.^8^ The result indicates that PtSe_2_, InSe, and SnSe may present higher electron currents, and SnSe could exhibit a high hole current. Experimentally, 2D InSe FETs have recently demonstrated a record high transconductance and a room-temperature ballistic ratio, surpassing the performance of state-of-the-art Si FETs.^9^ However, the air stability for device fabrication and maturity in obtaining large-area growth may require more research efforts for further verification. The simulation also shows that WS_2_ contains properties for both nFET and pFET applications due to its low mass and high phonon mobility, but it faces problems related to dielectric effects and contact resistance for its large bandgap. Thus, further material synthesis and characterization are needed to address the limitations. Given these challenges, the intensive growth efforts using MoS_2_ and WSe_2_ have positioned them as the most popular n- and p-channel materials in many recent device reports, underscoring their potential to meet current technological demands.

**Table 1:** **Basic Parameters of Monolayer MoS**_**2**_**and WSe**_**2**_^a^

2DM parameters	MoS_2_ nFETs	WSe_2_ pFETs
Lattice constant (*a*/*c*) [Å]	3.16/3.172	3.286/3.376
Band-gap [eV]	2.4	2.01
Electron affinity [eV]	4.03	3.37
Dielectric constant (ε)	4.8	4.5
Mobility [cm^2^/V·s]	217	126
Effective mass *m*_e_ [*m*_0_]	0.5726	0.3450
Effective mass *m*_h_ [*m*_0_]	0.6591	0.3445
Contact resistance [Ω·μm]	123	750

a Material parameters of n-type MoS_2_ and p-type WSe_2_ were used in the device simulations, where *m*_0_ is the free electron mass. Reproduced with permission from ref (2). Copyright 2024 Springer Nature Ltd.

### 2.2 Materials Synthesis

**Figure 3:**
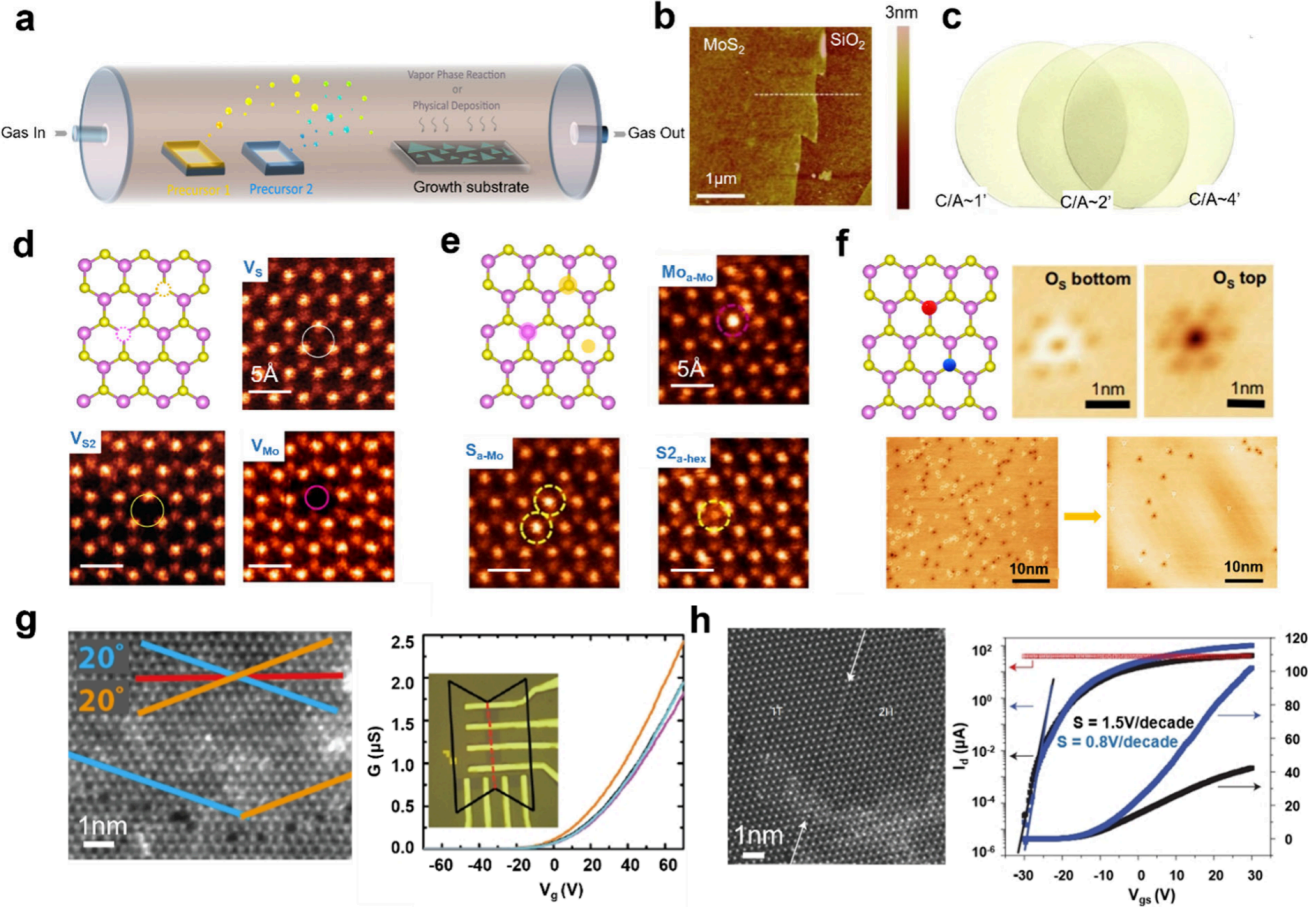
**Growth and defect engineering of 2D materials. a.** CVD growth of TMDs. Reproduced with permission from ref (12). Copyright 2021 Springer Nature Ltd. **b.** AFM image of a monolayer MoS_2_ film on a SiO_2_/Si substrate. Reproduced with permission from ref (11). Copyright 2022 John Wiley and Sons. **c.** Wafer-scale monolayer MoS_2_ films grown on 2-in. *c*-plane sapphire substrates with different off-cut angles. Reproduced with permission from ref (16). Copyright 2023 Springer Nature Ltd. **d.** HAADF-STEM images of V_S_, V_S2_, and V_Mo_ defects in monolayer MoS_2_. Reproduced with permission from ref (17). Copyright 2013 American Chemical Society. **e.** HAADF-STEM images of Mo and S adatoms in monolayer MoS_2_, including Mo adsorbed on top of the Mo site (Mo_a-Mo_), single S atoms adsorbed on Mo sites (S_a-Mo_), and disulfur interstitial in the center of the hexagonal ring (S2_a-hex_).^17^ Reproduced with permission from ref (17). Copyright 2013 American Chemical Society. **f.** STM images of S dopants in CVD and OHVPD WS_2_. Reproduced with permission from ref (14). Copyright 2022 Springer Nature Ltd. **g.** Left: HAADF-STEM image of a single-layer MoS_2_ film displaying a ±20 degree mirror twin boundary. Right: Linear transfer curves of four FETs, derived from a single mirror twin MoS_2_ crystal, representing both pristine (magenta and cyan) and grain-boundary-included (black and orange) crystals, with the grain boundaries aligned either perpendicular or parallel to the electron flow (right). Reproduced with permission from ref (24). Copyright 2013 Springer Nature Ltd. **h.** Left: HAADF-STEM image of an atomically thin phase boundary (indicated by the arrows) between the 1T and 2H phases in a monolayer MoS_2_ nanosheet. Right: Transfer characteristics of a bottom-gated device, measured at 1 V V_ds_. Blue and black curves represent devices with 1T phase electrodes and 2H phase, respectively. The red curve indicates the 1T channel device’s current, demonstrating its metallic character. Reproduced with permission from ref (25). Copyright 2014 Springer Nature Ltd.

The early stages of 2D FET research predominantly relied on mechanical exfoliation methods.^10^ However, this limits the scalability and manufacturing applicability. Adopting wafer-scale synthesis techniques becomes crucial to implement large-scale integration of 2D FETs. Among the approaches, chemical vapor deposition (CVD) is the most popular and efficient method for growing 2D materials. In 2012, researchers first synthesized micron-sized monolayer MoS_2_ single crystals using vapor phase CVD.^11^ A molybdenum oxide (MoO_3_) solid precursor and a sulfur powder source were placed in a tube furnace, which was heated to high temperatures, causing the MoO_3_ to react with the sulfur vapor and form MoS_2_ on sapphire substrates (Figure 3, a and b).^12^ Subsequently, other 2D materials, including MoSe_2_, WS_2_, and WSe_2_, have been synthesized via CVD with solid precursors, where the growth process requires precise control of temperature, pressure, precursor concentration, and growth time. For improving the large-area coverage^13^ and uniformity of the 2D films, there has been a push to migrate from vapor phase CVD to metal–organic chemical vapor deposition (MOCVD),^14^ which has become a critical research area for industry applications. In addition to the requirement of uniformity, single crystallinity (grain-boundary-free) is also needed for high-performance scalable devices. The typical approach is to ensure the single orientation of multiple seeds in the early stage of the 2D material growth, where recent studies have clarified that the orientation can be controlled by either substrate atomic structures through van der Waals (vdW) epitaxy or the atomic step edges via edge-guide growth.^15^Figure 3c demonstrates that wafer-scale homogeneous MoS_2_ monolayer films can be grown on different off-cut angle substrates.^16^ Separate from single crystallinity, the defect density in 2D films also needs to be lowered. It was found that the hydroxide vapor phase deposition (OHVPD) method, which utilizes tungsten hydroxide as a precursor, enables efficient sulfurization and results in a significantly lower defect density in WS_2_ monolayers.^14^ Considering all various factors, a proper growth tool for delivering uniform, low-defect-density, wafer-scale, and single-crystalline 2D materials is urgently needed and yet to be developed.

### 2.3 Characterization of Materials

In perfect crystal structures, the precise arrangement of the atoms forms a regular and periodic lattice. However, the reality of crystallinity reveals imperfections born out of the influence of entropy and synthesis-related factors. Due to the ultrathin structure of 2D materials, atomic characterization techniques are commonly applied, such as scanning transmission electron microscopy (STEM), scanning tunneling microscopy (STM), and atomic force microscopy (AFM), to assess the quality and defects of materials.^17,18^ Point defects in 2D materials can be classified into three types: vacancies, adatoms, and dopants. Chalcogen vacancies (V_X_) are frequently observed and are energetically stable. They create in-gap states near the conduction band edge (CBE) within the material’s bandgap (*E*_g_). On the other hand, transition metal vacancies (V_M_) are less common since they have higher formation energy, leading to more complex in-gap states and varied doping characteristics. Figure 3d presents the atomic-resolution high-angle annular dark-field (HAADF) images of single (V_S_) and double (V_S2_) S vacancies and a Mo vacancy (V_Mo_) in a MoS_2_ monolayer. Both V_S_ and V_S2_ introduce unoccupied deep levels (∼0.6 eV below conduction bands minimum) based on density functional theory (DFT) calculations, potentially acting as compensating centers in n-type MoS_2_.^17^ Comparably, adatoms can also exist in either transition metal (M) or chalcogen (X) elements, as shown in Figure 3e. An increase in the atomic number of the X element causes the valence band edge (VBE) to rise, leading to a smaller energy *E*_g_. Yet, a higher concentration of M atoms results in a higher CBE and a larger *E*_g_.^19^

It is noteworthy that, in 2D materials, chalcogen vacancies such as V_S_ are often substituted by environmental oxygen (O), which passivates the charged chalcogen vacancies. The STM images in Figure 3f identify the position of the O replacement in the upper and lower parts of monolayer WS_2_. The electrical performance illustrates that low O-substituted WS_2_ exhibits a significantly higher *I*_on_ and *I*_on_/*I*_off_ current ratio.^14^ Thus, material synthesis generally aims to reduce point defects to achieve a natural performance and avoid unwanted substitution effects. On the other hand, some dopants affect the electrical properties of host materials as foreign atoms replace M or X atoms in the lattice. For example, substituting Mo atoms with Group 5 elements, such as Nb or V, changes MoS_2_ from n-type conductivity to p-type, as these dopants have one less valence electron than Mo.^20^ Conversely, Group 7 dopants, such as Re or Tc, maintain n-type doping by creating donor levels near the CBE.^21,22^

Besides point defects, edge termination, grain boundaries (GBs), and phase transitions are often observed to influence lattice symmetry and electrical properties. In 2H-MoS_2_, carrier mobility is decreased by midgap boundary states originating from GBs comprising 7-5 and 8-4-4 membered rings, which causes increased carrier scattering.^23^ Meanwhile, mirror twin boundaries have minimal influence on carrier mobility in FETs, and a marginal increase in conductivity is observed when the device fabrication is aligned parallel to the grain boundary, indicating the importance of GBs when engineering large-area heterostructures (Figure 3g).^24^ Furthermore, electronic properties can be manipulated by inducing phase transitions. Figure 3h shows the 1T/2H heterostructure MoS_2_ achieved using an organolithium chemical method.^25^ The semiconducting 2H phase offers higher carrier mobility due to its strong in-plane bonding and well-defined bandgap, whereas the metallic 1T phase exhibits lower mobility and increased scattering from a distorted atomic arrangement (Figure 3h). In brief, elucidating the atomic structures and defects in 2D materials is vital for establishing their correlation with electrical properties, thereby facilitating the optimization of material synthesis and the realization of high-performance devices.

## 3 Key Factors in 2D-Based Transistors

**Figure 4:**
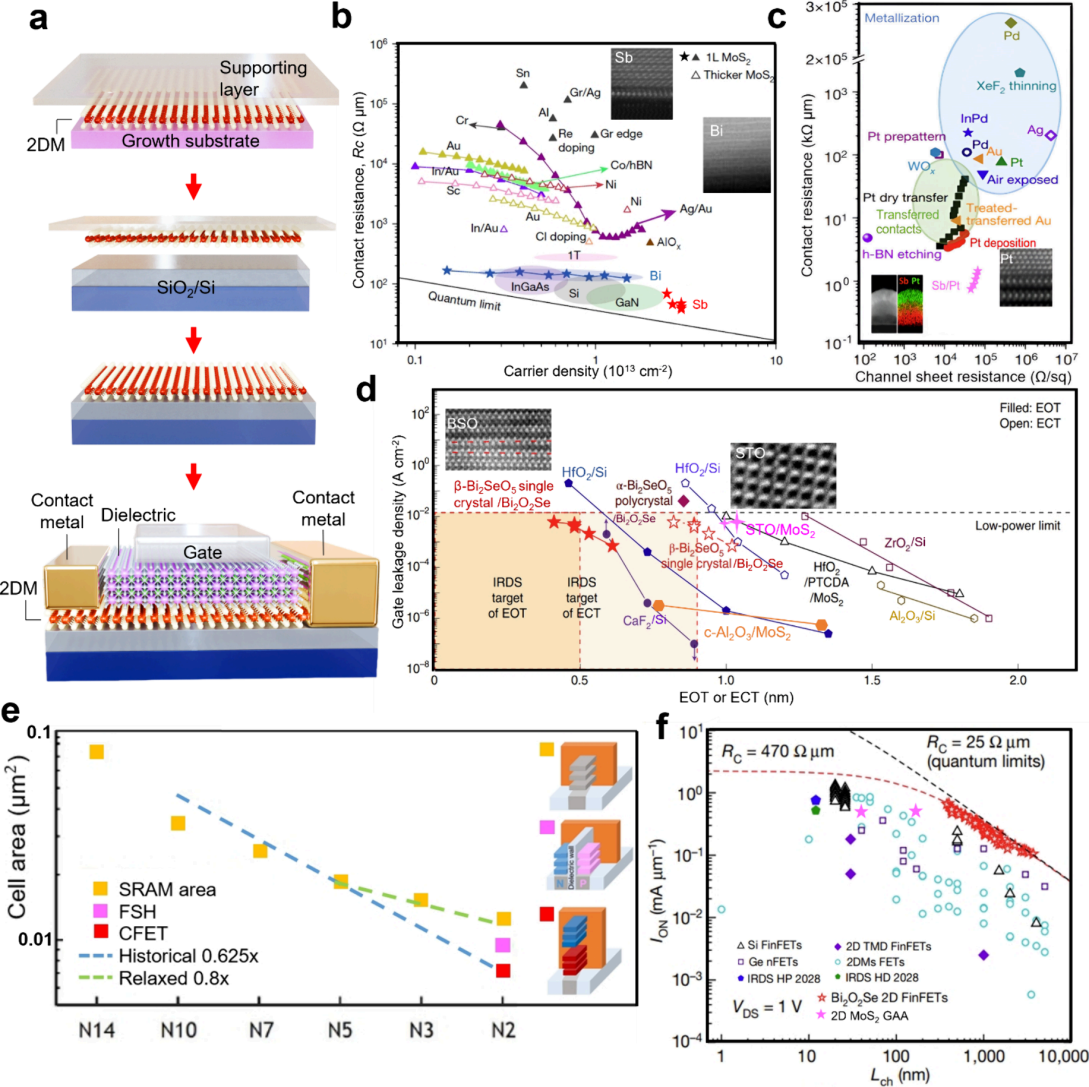
**2D-based transistors. a.** Schematic diagram of material transfer process and a FET featuring a single-layer 2D semiconductor. **b.** State-of-the-art contact technology for MoS_2_ transistors plotted as a function of *n*_2D_, showing the respective *R*_c_ of various semiconductor technologies (Si III–V and MoS_2_ with various layer numbers). Reproduced with permission from refs (30 and 31). Copyright 2021 Springer Nature Ltd. **c.** Contact resistance (*y* axis) and channel sheet resistance (*x* axis) of WSe_2_ FETs using different methods. Reproduced with permission from refs (32 and 33). Copyright 2022 Springer Nature Ltd. and IEEE. **d.** Comparison of different dielectrics in terms of EOT or ECT versus leakage current density. Reproduced with permission from refs (37, 38, and 41). Copyright 2022 and 2024 Springer Nature Ltd. **e.** Cell area scaling trends across technology nodes from N14 to N2. The graph shows a decrease in SRAM area, with historical scaling at 0.625× indicating significant area reduction per node, culminating in the CFET data point at N2. The relaxed scaling at 0.8× reflects a more cautious approach, leading to the SRAM point at N2, while the FSH point represents an alternative memory technology contributing to area reduction.^42^**f.** On-state current (*I*_ON_) versus *L*_ch_ of 2D FinFETs with commercial Si FinFETs, Ge n-type FETs (nFETs), 2D MoS_2_ FinFETs, 2DM FETs including MoS_2_, WS_2_, and WSe_2_, and 2D MoS_2_ GAA. Reproduced with permission from refs (43−45). Copyright 2023, 2022, and 2021 Springer Nature Ltd. and IEEE.

The performance and scalability of 2D-based transistors are influenced by several critical factors, including channel properties, contact resistance, dielectric scaling, and device architecture (Figure 4a). The quality of the channel material directly determines the performance of the device. Single-crystal 2D materials, typically grown on sapphire or metal substrates, must be transferred to the target substrate for device fabrication. Several transfer techniques have been developed to facilitate this process.^26^ However, most methods are currently limited to 6–8 in. To fully leverage the potential of 2D materials in electronics, there is a pressing need for the development of new facility designs and setups that can accommodate larger sizes and improve the scalability.

### 3.1 Contact Resistance

Contact resistance (*R*_c_) is a critical factor in the performance of 2D material devices, as it significantly affects carrier injection and, consequently, the overall device efficiency. The primary cause of high *R*_c_ is the presence of a Schottky barrier (SB) which forms between the metal electrode and semiconductor,^27^ resulting from the energy difference between the metal’s work function and the semiconductor’s electron affinity. Unfortunately, the SB between typical metals and 2D semiconductors is not tunable owing to the presence of metal-induced gap states (MIGS),^28^ which leads to the notorious Fermi-level pinning, and thus there is always an obvious SB. Various strategies have been used to tackle the issue, including transferring metal electrodes, optimizing metal evaporation, and imposing heavy doping in 2D materials. Figure 4b summarizes the *R*_c_ values of different materials. Recent advancements in transfer techniques have further enhanced the integration of metals with 2D materials, allowing for the preservation of their intrinsic properties during contact formation.^29^ Methods such as low-temperature or buffer-layer assisted metal deposition also have demonstrated the ability to create high-quality vdW contacts, minimizing damage and optimizing performance.^30−32^ One important breakthrough in n-type contact was achieved by depositing semimetal bismuth (Bi) onto monolayer MoS_2_, resulting in low-resistance (“ohmic”) contacts of 123 ohm micrometers.^30^ The semimetal–semiconductor contacts with a near-zero density of states at the Fermi level of a semimetal help to avoid gap-state pinning, which reduces MIGS and allows for gap-state saturation. Similarly, Sb is also an ideal n-type contact metal for 2D materials, reaching an ultralow *R*_c_ of 42 ohm micrometers.^31^

However, p-type contact is more challenging since the high-work-function metals require high sublimation energy. Figure 4c shows the performance of WSe_2_ FETs with different methods. Through industrially compatible electron-beam evaporation, integrating high-work-function metals (Pd, Pt) with MoS_2_ and WSe_2_ has given p-type devices with the lowest *R*_c_ of about 3.3 kilo-ohm micrometers.^32^ Additionally, by utilizing multiple approaches, including optimizing different Sb/Pt contact compositions and incorporating advanced oxide-based encapsulation and doping techniques, WSe_2_ FETs achieved a record-low pFET contact resistance of 0.75 kilo-ohm micrometers, setting a record benchmark for monolayer 2D pFETs.^33^ Despite these advancements, pFET contact resistance still remains significantly higher than that of nFETs, indicating the need for more research to improve p-type contact performance. Strategies like selective doping in the contact region and implementing edge-contact structures show promise in addressing these challenges by lowering the Schottky barrier and minimizing the Fermi level pinning, ultimately paving the way for more efficient and high-performing p-type 2D electronic devices. However, obstacles remain, particularly regarding doping stability and the trade-off between defect-induced mobility degradation and heteroatomic doping in the 2D lattice. Apparently, more research efforts are needed to match the p-type 2D transistors to the already high-current n-type 2D transistors.

Most of the current progress in 2D material devices has been focused on top contact configurations. However, edge-contact technology is becoming increasingly critical for advanced-node circuits, particularly for 2D channel GAA and CFET. Edge-contact devices for graphene have shown significant improvements in electrical performance, achieving low contact resistance and high room-temperature mobility, thereby enhancing the scalability of multilayered 2D material structures.^34^ Nevertheless, creating highly reproducible ohmic edge contacts remains a challenge. Developing techniques compatible with existing microelectronics processes, such as suitable etching and metal-filling strategies, is essential for ensuring the reliable formation of ohmic edge contacts.

### 3.2 Dielectric Scaling

Regarding gate dielectric scaling, a delicate equilibrium must be maintained between reducing the dielectric thickness to increase gate capacitance for better gate coupling and minimizing gate leakage currents. Apart from hBN, which is the most-used layered insulator but causes excessive leakage currents for 2D FETs,^35^ several insulators, including mica, HfO_2_, perovskites, or CaF_2_, exhibit lower leakage currents favorable insulating properties. Among them, high-dielectric-constant (κ) materials with a capacitance equivalent thickness (CET) less than 1 nm are highly desirable for future 2D MOSFETs. Due to the atomically smooth surface and absence of dangling bonds in 2D materials, atomic layer deposition often results in poor uniformity and leakage. Introducing interfacial layers, such as oxidized metal layers, organic molecules, or surface treatments, can enhance the dielectric uniformity. A monolayer molecular crystal, PTCDA, has been reported as a seeding layer, enabling the deposition of high-κ gate dielectrics with an effective oxide thickness (EOT) of nearly 1 nm.^36^ Separate from TMD transistors, through utilizing the epitaxial growth method to form vertical fin arrays on insulating substrates and a controllable oxidation process, the integration of 2D Bi_2_O_2_Se fins with a high-κ self-oxidized compound Bi_2_SeO_5_ heterojunction has been successfully achieved. The devices exhibit high performance and low power in terms of mobility (270 cm^2^/(V·s)), off-state current (1 pA/μm), and current on–off ratio (10^8^).^37^ Single-crystalline perovskite SrTiO_3_ has been discovered to have an ultrahigh κ value as a gate dielectric (Figure 4d). It produces an ideal subnanometer CET and a low leakage current of less than 10^–2^ A/cm^2^ at 2.5 MV/cm through integrating a vdW gap between the 2D channel and SrTiO_3_. Using SrTiO_3_, typical short-channel transistors based on a scalable CVD MoS_2_ film demonstrate a sharp subthreshold swing as low as 70 mV per decade, along with an on–off current ratio reaching up to 10^7^. Similarly, other high-κ materials, transferred on 2D channels to include a vdW gap, have also shown enhanced gate controllability.^38−40^ Recent research highlights the potential of atomically thin c-Al_2_O_3_ as a quality top-gate dielectric layer in 2D FETs. After epitaxial lift-off and intercalative oxidation techniques, the thin c-Al_2_O_3_ layer meets low-power device standards due to its optimal crystalline structure. Top-gate MoS_2_ FETs employing c-Al_2_O_3_ have demonstrated excellent electrical performance with a steep subthreshold swing, a significantly high on–off current ratio, and negligible hysteresis.^41^ Conclusively, while numerous methodologies enhancing gate controllability in 2D materials have emerged, the challenge remains to ascertain if these simultaneously meet the rigorous prerequisites of gate dielectrics, encompassing scalable deposition, minimal perturbation of 2D channels during integration, low equivalent oxide thickness (EOT), diminished leakage currents, elevated breakdown voltages, and superior reliability.

### 3.3 Device Architecture

As mentioned earlier, 2D semiconductors in scaled devices provide ultrathin body structures that help reduce short-channel effects. Using 2D materials also enables advanced transistor geometries like dual-gated, fin, and GAA designs. Figure 4e shows the cell area decrease as it varies with technology nodes on different architectures.^42^ For FinFETs, the standard cell scaling requires a reduction in fin count, which leads to reduced drive current strength and increased variability. Thus, the nanosheet architecture offers a solution by enabling a larger effective channel width through vertically stacking nanosheet-shaped conductive channels in single-fin standard cells. The forksheet (FSH) device architecture addresses the challenge of increasing the effective channel width in traditional nanosheet structures by introducing a dielectric wall between n- and p-MOS devices before gate patterning. To achieve the maximum effective channel width, the CFET architecture stacks n- and p-MOS components on top of each other, transferring the spacing between the n- and p-MOS to the vertical direction. Figure 4f illustrates the on-state current versus channel length for various FETs, including n-type 2D Bi_2_O_2_Se/Bi_2_SeO_5_/HfO_2_ FinFETs, n-type 2D MoS_2_ FinFETs, 2DM FETs with materials such as MoS_2_, WS_2_, and WSe_2_, encompassing both n-type and p-type configurations, 2D MoS_2_ GAA, as well as commercial Si FinFETs and Ge n-type FETs.^43−45^ Despite traditional Si and Ge FETs’ established performance, 2D FinFETs and 2D GAA exhibit superior mobility and drive current, making them strong candidates for next-generation transistors. Recently, CFETs based entirely on 2D materials have been realized, demonstrating their potential to enhance performance while reducing footprint.^46,47^ In summary, the device architectures have advanced from planar configurations to complex 3D stacks, transitioning from FinFETs to nanosheets, FSH, and CFETs. 2D materials, characterized by their absence of dangling bonds, exhibit substantial potential in these multilayered structures, contributing to improved performance and scalability. These advancements offer promising pathways for future transistor designs, emphasizing the significance of 2D semiconductors in continuing the scaling of electronic devices.

## 4 Benchmark Si and 2D Materials at Advanced Nodes

### 4.1 2D Materials Compared to Si at the Transistor Level

**Figure 5:**
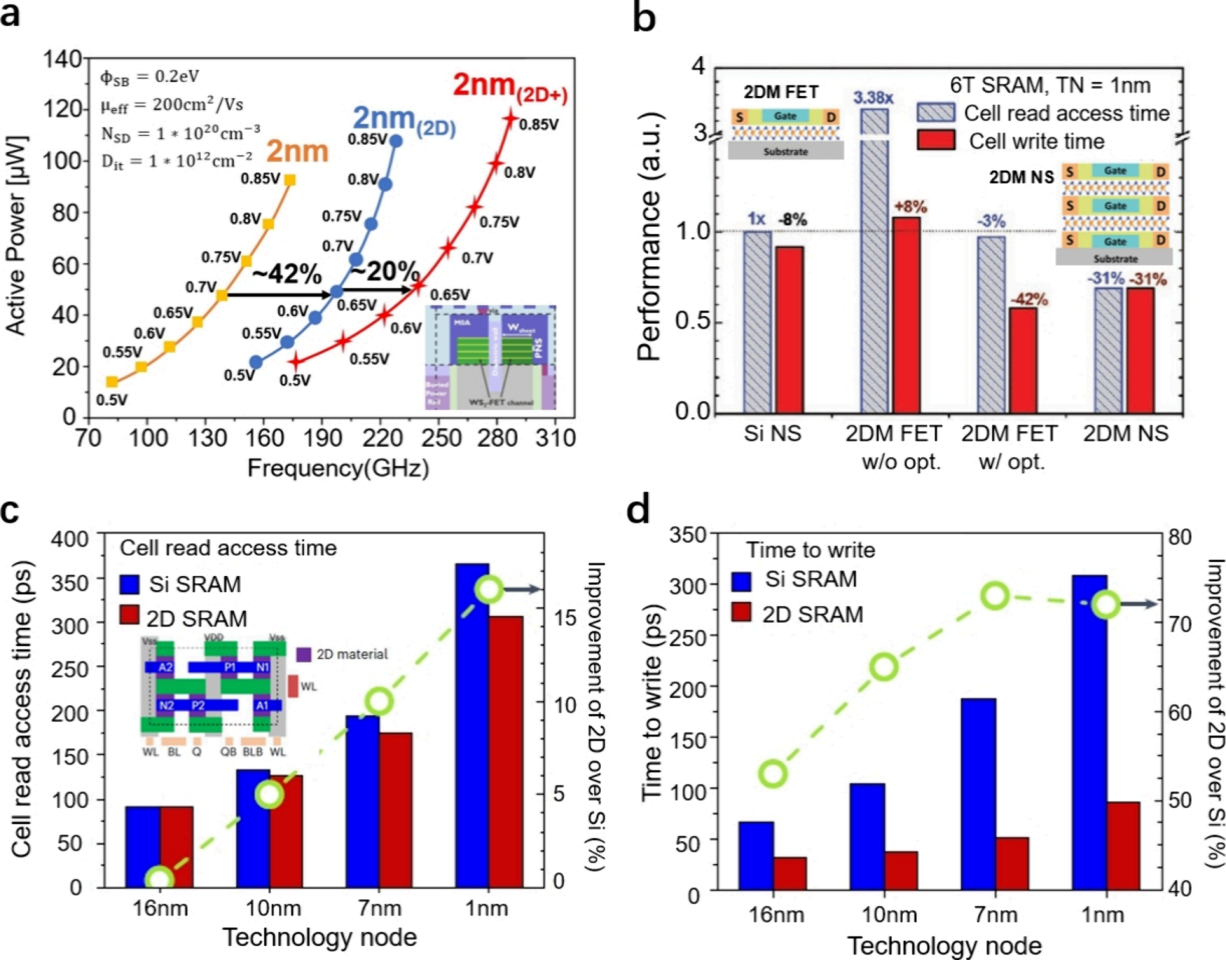
**Benchmark Si and 2D materials at advanced nodes. a.** Active power in relation to frequency for 2 nm_(Si-FSH)_, 2 nm_(2D)_, and 2 nm_(2D+)_ nodes in a 4-stack 1L WSe_2_ FET. A practical device mobility of 200 cm^2^/(V·s) is presumed. Contact gate pitch (CGP) scaling results in an 8% increase in area per node, with the same metal pitch and tracks assumed for the 2 nm node.^49^**b.** Performance comparisons among various the SRAM cells at TN = 1 nm at fixed leakage power. Reproduced with permission from ref (50). Copyright 2022 John Wiley and Sons. **c.** Comparisons of cell read access time between 2DM-based and Si SRAM cells as technology nodes scale from 16 to 1 nm. Reproduced with permission from ref (2). Copyright 2024 Springer Nature Ltd. **d.** Comparisons of cell write time between 2DM-based and Si SRAM cells as technology nodes scale from 16 to 1 nm. Both the cell read access time and write time increase as the supply voltage decreases and wire resistance increases with scaling. Reproduced with permission from ref (2). Copyright 2024 Springer Nature Ltd.

The main challenges for 2D materials compared to Si involve driving current limitations due to large extension and high contact resistance. However, studies show that thin 2D FETs provide better electrostatic control than Si NS FETs at a constant off-current, evaluating electrostatic control, subthreshold swing, and drain-induced barrier-lowering characteristics.^48^ Based on the Berkeley short-channel IGFET model for integrated multigate devices (BSIM-IMG) framework, a physics-based compact model for 2D FETs has been developed on WSe_2_ and validated experimentally with MoS_2_ devices.^49^ Ahmed et. al have demonstrated that a 40% performance increase is achieved when transitioning from a 2 nm Si-FSH node to a 2 nm 2D node. Additionally, with essential modifications to the architecture, vertical sheet pitch, and gate length scaling, advanced 2 nm (2D+) nodes enable a >20% performance gain at an iso-power condition of 0.7 V (Figure 5a).^49^

### 4.2 2D Materials Compared to Si at the Circuit Level

The final goal of using 2D materials in electronics is to incorporate them into integrated circuits (ICs). Figure 5b presents the performance comparisons among Si NS FETs, 2DM FETs without optimization, 2DM FETs with optimization, and 2DM NS FET static random-access memory (SRAM) cells at the 1 nm technology nodes. The optimized 2DM SRAM with an improved subthreshold swing exhibited a lower cell read access time (−3%) and write time (−42%) compared to those of the Si SRAM cell.^50^ A recent simulation framework incorporating front-end-of-line (FEOL) and back-end-of-line (BEOL) designs examined the potential of 2D material-based transistors at the circuit level. Calibrated MoS_2_ n-FETs and WSe_2_ p-FETs were utilized across various technology nodes to analyze the SRAM functionality. Results demonstrate superior stability, enhanced operating speed, and improved energy efficiency in 2D material-based SRAM circuits compared to Si-based SRAMs. Specifically, 0.4% to 16% reductions in cell read access time, 53% to 72% improvements in write time operations, and 36% to 60% lower dynamic power consumption were observed, particularly at the 1 nm node (Figure 5, c and d).^2^ Thus, integrating 2D materials into ICs holds promise for the future of electronics, as they demonstrate superior stability, enhanced operating speed, and significantly higher energy efficiency compared to Si-based transistors.

## 5 Conclusions and Perspectives

Undoubtedly, 2D materials, particularly TMDs, have shown great promise for next-generation electronics, thanks to their inherent advantages. The unique properties enable 2D materials to achieve high on–off current ratios, low subthreshold swings, and improved carrier mobility. As a result, they have outstanding prospects to deliver high-performance, low-power-consumption electronic devices that are compatible with BEOL processes. However, several technical challenges must be addressed to successfully integrate 2D materials into electronics. One key factor involves the large-area growth of high-quality 2D materials at 8- to 12-in. scales. Advancements in epitaxial growth methods are required to optimize the stoichiometry, chemical purity, and yield of the 2D materials. Further research is needed to establish baseline properties, layer control, and defect control. At the same time, reliable and efficient large-area material transfer technologies are crucial for device fabrication. Current techniques, such as wet transfer and electrochemical delamination, can introduce contaminants, wrinkles, or tears in 2D materials. Additionally, device-related issues in 2D electronics, including optimizing electrical contacts, refining doping techniques, fabricating high-quality p–n junctions, and investigating novel dielectric materials and interfaces, urgently need to be addressed. The ongoing development of electronics demands continued device scaling and improved performance. 2D materials hold the potential to bring transformative changes to the industry, introducing new applications in transistors, sensors, and flexible or wearable electronics. Despite the challenges, the diverse range of 2D materials is poised to pave the way for technological breakthroughs in the future.
